# HAE patient self-sampling for biomarker establishment

**DOI:** 10.1186/s13023-021-02021-x

**Published:** 2021-09-28

**Authors:** Toni M. Förster, Markus Magerl, Marcus Maurer, Selen Zülbahar, Susanne Zielke, Neil Inhaber, Donatello Crocetta, Arndt Rolfs, Volha Skrahina

**Affiliations:** 1 CENTOGENE GmbH, Rostock, Germany; 2 Dermatological Allergology, Allergie-Centrum-Charité, Department of Dermatology and Allergy, Charité - Universitätsmedizin Berlin, Berlin, Germany; 3 Takeda Pharmaceutical Company Limited, Lexington, MA USA; 4 Takeda Pharmaceuticals International AG, Zurich, Switzerland; 5 Universität Rostock, Medizinische Fakultät, Rostock, Germany; 6 Arcensus GmbH, Goethestrasse 20, 18055 Rostock, Germany

**Keywords:** Hereditary angioedema, Observational clinical study, Biomarker, Cleaved high molecular weight Kininogen, Self-sampling

## Abstract

**Background:**

Hereditary Angioedema (HAE) is a genetic disorder that leads to frequent angioedema attacks in various parts of the body. In most cases it is caused by pathogenic variants in the *SERPING1* gene, coding for C1-Inhibitor (C1-INH). The pathogenic variants in the gene result in reduced C1-INH levels and/or activity, which causes aberrant bradykinin production and enhanced vascular permeability. The standard-of-care diagnostic test is performed biochemically via measuring C1-INH level and activity as well as the C4 level. This, however, does not allow for the diagnosis of HAE types with normal C1-INH. There is an urgent need to identify and characterize HAE biomarkers for facilitating diagnostics and personalizing the treatment. The Hereditary Angioedema Kininogen Assay (HAEKA) study aims to measure the dynamics of cleaved High Molecular Weight Kininogen (HKa) and other metabolite levels during the angioedema and non-angioedema state of the disease. The metabolites will be analyzed and verified by liquid chromatography ion mobility high resolution mass spectrometry (LC/IM-QToF MS) of dried blood spot (DBS) cards upon the study completion. The study design is truly innovative: 100 enrolled participants provide blood samples via DBS: (1) every 3 months within 2 years during regular study site visits and (2) by at-home self-sampling during HAE attacks via finger pricking. We are presenting a project design that permits clinical study activities during pandemic contact restrictions and opens the door for other clinical studies during COVID-19.

**Results:**

As of October 2020, there are 41 patients from 5 sites in Germany enrolled. 90 blood samples were collected during the regular visits, and 19 of the participants also performed self-sampling during the HAE attacks from which a total of 286 attack blood samples were collected. Participating patients rate the study procedures as easy to implement in their daily lives. The concept of home self-sampling is effective, reproducible, and convenient especially in times of contact restrictions due to the COVID-19 pandemic.

**Conclusions:**

It is the hope that the HAEKA study will complete in 2023, reveal biomarker(s) for monitoring HAE disease activity, and may help to avoid HAE attacks via applying medication prior to the symptom onset.

**Supplementary Information:**

The online version contains supplementary material available at 10.1186/s13023-021-02021-x.

## Background

Hereditary Angioedema (HAE) is an autosomal dominant genetic disorder that affects 1 in 50,000 people [1–4]. HAE is caused in most cases by a pathogenic variant in *SERPING1,* encoding the C1-inhibitor (C1-INH), leading to deficiency or dysfunction of C1-INH (HAE types 1 and 2 [HAE-1/2], respectively). There are up to 748 different *SERPING1* variants reported so far [5]. Reduced levels of functional C1-INH, in HAE-1/2, lead to dysregulation of the contact system with cleavage of High Molecular Weight Kininogen and increased bradykinin production, causing enhanced vascular permeability and swellings. Hence, HAE patients experience unpredictable episodes of angioedema attacks of extremities, airway, face, and the gastrointestinal tract in addition to the risk of death in the case of untreated laryngeal attacks.

Additional forms of HAE with very similar clinical phenotypes exist, all of them independent of *SERPING1* pathogenic variants and hence with normal C1-INH levels (nC1-INH-HAE). The increased availability of genetic analyses has uncovered pathogenic variants in genes coding for various components of the fibrinolytic and contact system pathways in families with recurrent angioedema attacks: *FXII*, *ANGPT1*, *PLG1*, *MYOF*, *KNG1* or *HS3ST6* [6–11]. However, the HAE diagnostic work up currently recommended by international guidelines only calls for the biochemical measurement of C1-INH level and activity and complement component 4 (C4) level, which allows for diagnosing HAE-1/2 but not nC1-INH-HAE. Guidance on the diagnostic work up in patients suspected to have nC1-INH-HAE is needed including recommendations on when to perform genetic analyses and which ones [12].

In order to guide the therapeutic management in HAE, it will be of great benefit to utilize biomarkers, i.e. biological indicators of disease state, as it may be possible to personalize the treatment according to the biomarker status. In general, biomarkers are used to: (a) diagnose a disease, (b) evaluate the response to therapies, (c) make predictions regarding the clinical course of the disease, and (d) evaluate disease severity and activity. The latter points may be an advantage for HAE patients: e.g. a prognostic HAE biomarker could be used by patients to monitor their disease progression so upcoming attacks can be predicted and treated early enough to prevent them. An HAE biomarker that fulfills all of these criteria has not yet been identified. C1-INH and C4 are used as diagnostic biomarkers [13], however they have no prognostic value and fail to diagnose nC1-INH-HAE. Among other molecules under consideration as prognostic biomarkers are bradykinin, plasma kallikrein, Factor XII, and cleaved high molecular weight kininogen (HKa) [13]. To date, none of these biomarker candidates has been validated.

A recent report describes a novel 2D-LC–MS/MS method to measure HKa with the ability to successfully distinguish HAE patients from control groups [14]. Another paper has shown that HKa levels are indeed higher during acute attacks of HAE patients and that HKa correlates with the activity of the disease [15]. With these recent advances in mind, we will further verify HKa as a suitable biomarker for HAE and validate its clinical and prognostic value, by using approaches that helped with the development of biomarkers for other rare diseases, e.g. Gaucher disease, Niemann-Pick-C disease, Fabry disease, and Farber disease [16–19]. In contrast to the approach reported by Zhang et al., we will not use frozen plasma samples, but rather establish a method combining finger pricking, DBS filter CentoCard® technology, and a validated LC/IM-QToF MS technique. Moreover, by applying an untargeted metabolomics approach, we will analyze the blood samples for further potential biomarker(s). Screening the entire metabolome may identify biomarker molecules that are not part of the obvious C1-INH pathways, but that are linked to the HAE-pathophysiology in a more complex way.

Here, we report on the aims, setup and first results of the Hereditary Angioedema Kininogen Assay (HAEKA) study (ClinicalTrials.gov registry number: NCT04091113), which aims to validate HKa as a diagnostic and prognostic biomarker for HAE disease and to identify additional ones. To this end, the HAEKA study uses a seminal concept: participating patients perform self-sampling at home during HAE attacks using kits that contain safety lancets and CentoCard®(s) for blood collection. This way, it is possible to monitor the metabolites in the blood also during the course of an HAE attack. This study design was never applied before in HAE studies.

## Results

### Cohort description

**Fig. 1:**
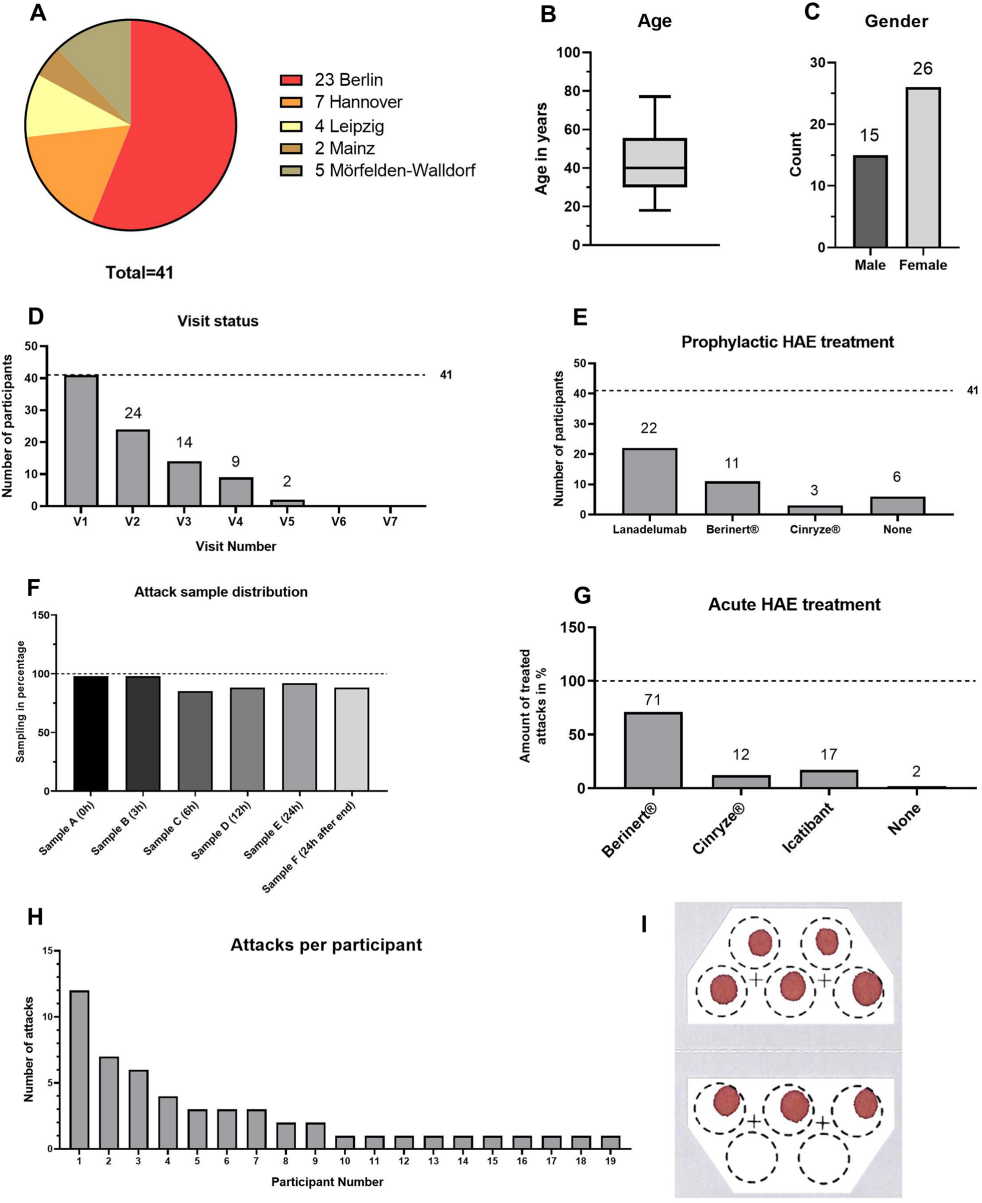
Recruitment details of the HAEKA study as per 07.10.2020. **a** Number of participants enrolled per study site. **b** A boxplot displaying the age distribution of the participants. **c** Gender distribution of the participants. **d** Visit status of all participants in the HAEKA study. The number of completed Visits 1–7 is shown. **e** Prophylactic treatment of HAEKA participants: it is shown how many participants take each respective medication (one subject received both Lanadelumab and Berinert®). **f** An overview showing how often the different samples **a**–**f** of each attack have been collected. **g** Acute treatment of HAE attacks: it is shown how many percent of the participants take a respective medication to treat an attack. **h** Overview on the amount of documented attacks per participant. **i** Exemplary CentoCard® showing the quality of participant’s self-sampled blood spots

The HAEKA study started the patient enrolment in November 2019. As of 07.10.2020, seven study sites in Germany have been initiated (see Additional File 1) and 41 participants were enrolled by five of these sites (Fig. 1a).

The age distribution in Fig. 1b shows that the median age is 40, the youngest participant is 18 years old and the oldest participant is 77 years old. 15 of the participants are male (37%) and 26 participants are female (63%) (Fig. 1c).

Every enrolled participant completed the visit 1 and follow up visits were completed by a decreasing number of participants according to their enrollment date: 24 × Visit 2, 14 × Visit 3, 9 × Visit 4, and 2 × Visit 5 (Fig. 1d).

The overall number of documented attacks by all participants is 52. Participants collected 286 samples out of a maximum of 312 samples (six samples per attack), which shows a sampling success of 92% during the attacks. Sample A (beginning of the attack) was collected in almost all cases, whereas the most difficult sample to collect was sample C (6 h ± 2 h after beginning of attack, documented in 44 attacks out of the 52 attacks in total) (Fig. 1f).

Less than half of the enrolled participants (19) contributed with sampling during attacks (Fig. 1h). The most active participant sampled 12 attacks by him/herself, whereas most participants (10/19) sampled only one attack so far. Of the 19 participants contributing with attack samples, six were receiving prophylactic therapy with Lanadelumab. Overall, the quality of the self-sampled dry blood spots on the CentoCard® is sufficient and laboratory analyses can be performed accordingly (Fig. 1i).

Lanadelumab treatment resulted to be the most commonly used prophylactic therapy among the HAEKA participants and it is distributed in a balanced way: 22 participants receive Lanadelumab and 19 do not (Fig. 1e). The plasma derived C1-INH is used by 14 participants, respectively, whereas six participants do not use any prophylactic medication. There was only one documented attack that was not treated with acute treatment (Fig. 1g). All other documented attacks were treated either with Berinert® (71%), Icatibant (17%), or Cinryze® (12%).

### Participant contact

**Fig. 2:**
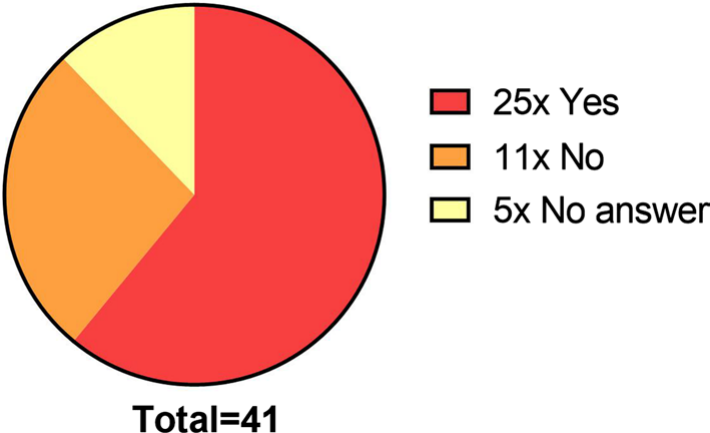
Response of the HAEKA participants for being contacted by the study nurse

Among all enrolled HAEKA participants, 25 patients consented to being contacted by the HAEKA study nurse (61%, Fig. 2). Many of the follow-up visits were organized from home, so the participant did not visit the treating physician. Instead, the HAEKA follow-up box was sent directly to the patient and the follow-up visit was performed by the participant itself, with the support of the study nurse via phone. This procedure also helped to complete all follow up visits on time.

CENTOGENE conducted a participant survey by asking questions about the study feasibility and convenience. All 25 participants having agreed to be contacted by the study nurse were asked to participate in the survey. 18 participants took part and the overall results are shown in Table 1. The explanation and understanding of the HAEKA procedures were rated as very good by the participants, in a scale of 1–5 (1: very good, 5: not acceptable). Also, the pain level experience by the usage of lancets was mostly rated to be not painful and the process of blood application to the CentoCard® was found to be easy. No participant has reported any side effects after finger pricking. In summary, the HAEKA study had a low influence on the daily life of the participants and is well accepted.

**Table 1:** Survey for HAEKA study convenience on 18 participants

How do you evaluate	1 Very good	2	3	4	5 Not acceptable
The content of the HAEKA Study Box	18x				
Understanding of the description of study procedures	18x				
Understanding of the arrangement of the HAEKA Study Box	18x				
The level of pain associated with use of the safety lancets	6x	9x		3x	
Performing DBS sample collection via safety lancets	2x	13x		3x	
The impact of the study on your daily life	7x	11x			
Overall opinion	18x				

### Genetics of the participants

All of the 41 enrolled HAEKA participants underwent genetic confirmation of HAE-1/2 by the analysis of the blood sample collected during Visit 1. Table 2 summarizes the genetic variants that were found in the *SERPING1* gene. All identified variants show a heterozygous pattern and are classified as Class 1 – pathogenic according to the recommendations of American College of Medical Genetics (ACMG).

**Table 2:** Variants in the gene *SERPING1*, detected in the HAEKA study participants as per 07.10.2020

Variant	Frequency	Literature
c.1450C > T	3	[20]
c.686-3C > G	1	[21]
c.52-1G > A	1	[22]
Heterozygous deletion encompassing exons 3–4	2	[23]
c.550G > A	3	[24]
c.1346 T > G	1	[25]
c.358_377dup	1	[22]
c.551-2A > G	1	[22]
c.896G > A	1	[25]
c.1250-2A > G	2	[22]
c.329_341del	2	[22]
c.1480C > T	3	[26]
Heterozygous deletion encompassing exon 8	2	[22]
c.1250-1G > A	1	[27]
Heterozygous deletion encompassing exons 5–6	1	[28]
Heterozygous deletion encompassing exons 1–6	1	[29]
c.1396C > G	1	[22]
c.1372G > A	1	[30]
c.51 + 3A > G	2	[21]
c.467C > A	1	[22]
c.1397G > A	4	[31]
c.1030-1G > C	1	[26]
c.1408dup	1	[32]
c.1333_1336delACAG	1	[32]
c.1263_1264dup	1	[32]
c.1478G > T	2	[32]
Total	41	

All variants are classified as Class 1—pathogenic and are heterozygous

A total of 26 different pathogenic variants were detected in the 41 HAEKA participants. All of the variants were previously reported in the literature. Whereas most of the variants were detected only once, the most frequent variant (c.1397G > A) was detected in four participants. These four cases are coming from one center but from two separate families, each time represented by a child and a mother. In contrast, the point mutations c.1480C > T and c.1450C > T are detected three times each in independent participants.

## Discussion

The HAEKA study is running for approximately one year and 41 of the planned 100 participants were enrolled. All of the enrolled participants were confirmed to have HAE-1/2 by sequencing the *SERPING1* gene. All genetic variants in the HAEKA participants were already described in the literature (Table 2).

The HAEKA study is enrolling HAE patients with different kinds of prophylactic and on-demand treatments and is collecting samples both before and after initiation of Lanadelumab (if applicable) and in the course of HAE attacks. The choice of HAE therapy is solely at the discretion of the treating physician, therefore, HAEKA study is purely an observational study. Both HAE therapies and the onset of attacks have the potential to affect HAE biomarkers. Treatment diversity is important to explore effects on biomarkers and metabolites and serves as an important quality control for the measures (Fig. 1e, g). The most commonly used medication for treating acute attacks is Berinert® (used for treatment of 37/52 attacks) and the most commonly used prophylactic treatment is Lanadelumab (used by 22/41 participants).

The amount of blood samples collected by the patients during the attacks and the quality of the samples is excellent. Evaluation of the received CentoCard® from the home sampling via lancets shows that in all cases enough blood was collected in an acceptable quality (Fig. 1i). Approximately half of the enrolled participants (19) documented at least one attack (Fig. 1h). It is likely that the administration of prophylactic HAE therapy in many participants (Fig. 1e) has reduced the occurrence of attacks. It is especially gratifying that the participants were able to collect 92% of the possible samples during the attacks (Fig. 1f) in spite of the long time window allowed (0–24 h) and time points for sampling might often interfere with the work or sleep routine. We are thus glad about the motivation of the participants and their determination to take actively part in the study. We surely owe this success also to the ease of sample collection and the successful educational efforts, confirmed by the patient survey (Table 1). We conclude that self-sampling via safety lancets is safe, reproducible, stable and easily doable for patients.

The HAEKA study was designed to make the study activities as convenient as possible for the participants. By preparing boxes with all the necessary equipment and providing detailed explanations with brochures and videos, the effort for participants is kept at minimal. The home-sampling via the DBS CentoCard® provides high flexibility both to the participants and to the study site. The idea of home-based finger pricking in studies is not new and was for example successfully tested in a study by Pichler et al*.* for investigating coeliac disease [33]. The concept of self-sampling at home was especially fitting perfectly in times of contact restrictions due to COVID-19, reducing the impact of such restrictions in 2020. We propose the self-sampling as an important element to successfully arrange observational studies in times of COVID-19.

While self-sampling concept made it possible to conduct the HAEKA study during the COVID-19 pandemic, it also enables for the first time in HAE research to analyse real attack-derived blood samples from a large cohort. The second innovation is the coupling of these DBS samples with an untargeted metabolomic analysis by LC/IM-QToF MS. Other publications confirmed the valid approach of combining the home-based blood collection via lancets with the analysis via DBS Filtercard technology and/or Mass spectrometry [34, 35]. However, to our knowledge self-collected DBSs for metabolomic analyses were not described before, making it possible to get individual molecular fingerprints of the HAE edema states.

Eventually, the analysis of the samples collected in the HAEKA study may lead to the identification of novel metabolites that can be used as prognostic or diagnostic biomarkers for HAE. The need for HAE biomarkers that are associated with the frequency of attacks and the clinical course, progression, and response to therapy is described in detail in the review by Kaplan and Maas [13] and may lead to identification of important features of the pathophysiology of HAE. It summarizes possible candidates for such biomarkers, especially those being a part of the pathways with a direct C1-INH contribution. One of the such biomarker candidates is HKa, the cleaved by-product generated when its zymogen, high-molecular weight kininogen (HK), is processed by the protease kallikrein to generate the vasoactive peptide bradykinin and HKa.

## Conclusions

The HAEKA study is planned to be finalized in 2023 with 100 participants having completed the 2-year follow-up phase. The innovative study design of the HAEKA study enables new possibilities for the HAE research. By sampling the course of HAE attacks combined with a systematic, exploratory, untargeted metabolomics approach, we hope to unravel the yet unidentified biomarkers. It is expected that the analysis of these data will reveal new insights into the pathophysiology of HAE attacks.

## Study protocol and methods

### Eligibility criteria

The HAEKA study is a nation-wide, observational, longitudinal study being conducted in Germany. One hundred HAE-1/2 patients will be enrolled. The inclusion criteria are as follows:

(1) Informed consent is obtained from the participant. (2) The patient is diagnosed with HAE-1/2 based on international guidelines. (3) The patient has experienced ≥ 4 HAE attacks within the last 12 months before enrolment in the study. (4) The participant is older than 18 years old.

There are no specific exclusion criteria.

Participants receiving prophylactic, on-demand, and no treatment at all can be included in the HAEKA study. The study aims to include approximately 100 HAE patients stratified by prophylactic treatment with Lanadelumab or not in a 1:1 ratio. By including the different treatment groups, it will be possible to assess the effects of different treatments on the potential biomarkers.

### Study design

**Fig. 3:**
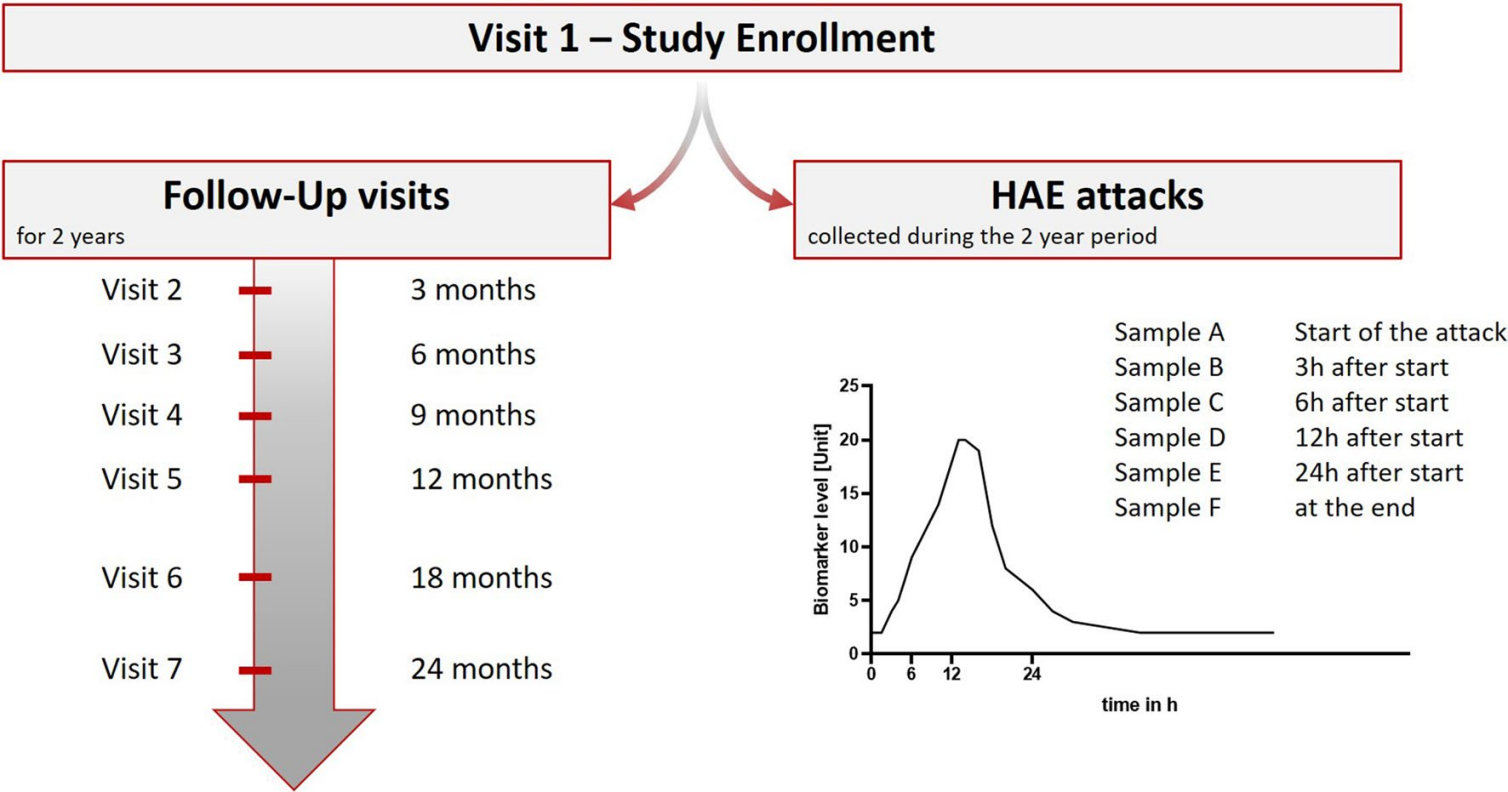
HAEKA Study sampling scheme. The left part shows in which intervals the 7 regular visits of each participant take place. The right part shows at which time points of an acute attack HAEKA participants shall collect samples **a**–**f** via finger pricking. The graph shows a hypothetical curve of a potential biomarker that reflects the course of an HAE attack

All enrolled participants will be monitored for a period of 24 months. Samples will be obtained during seven scheduled visits with three-month intervals in the first 12 months (visit 1–5) and six-month intervals thereafter (visit 6–7) (Fig. 3). At each routine visit, a Case Report Form will be completed (see next chapter) and a DBS Filtercard (CentoCard®) sample will be obtained. Each sample will be used for biochemical analyses (antigenic C1-INH, C4, and HKa levels), and the blood sample of visit 1 will undergo genetic analyses in order to confirm a HAE-1/2 diagnosis. If this diagnosis cannot be confirmed, the patient will be excluded from HAEKA.

Apart from the routine visits, every patient will be asked to collect samples individually by finger pricking (and apply to the CentoCard®) at the following time points during an HAE attack, should they occur (see Fig. 3):

- At the beginning of the edema attack: time point – 0
- 3 h ± 1 h (after the beginning of the attack)
- 6 h ± 2 h (after the beginning of the attack)
- 12 h ± 2 h (after the beginning of the attack)
- 24 h ± 4 h (after the beginning of the attack)
- At the end of the HAE attack (24 h after the termination of symptoms). This is the last blood draw obtained from the patient, e.g. if the attack lasts less than 3 ± 1 h only two blood samples will be collected.

By sampling blood at various time points during the attack, it may be possible to track the kinetic course of HAE pathophysiology by measuring potential HAE biomarkers. A metabolomics approach via Liquid Chromatography and mass spectrometry will unravel more insights into the molecular details of the HAE attacks (See the later chapter in Methods for details). To reduce the burden to the patient during the finger pricking, atraumatic safety lancets are used. These disposable consumables are established devices that enable a convenient sampling while causing minimal pain and effort for the patient (e.g. used routinely by diabetic patients). Each patient will receive “HAEKA-Boxes” that contain all the materials necessary for the sample collection, such as lancets, disinfection pads, and CentoCard®(s). The sampling procedure is explained thoroughly with educational materials for patients including both handouts and video material (https://www.centogene.com/haeka.html).

In case of any questions, participants can get in contact with the HAEKA study nurse. The study nurse can assist with the sampling procedure and the CRF completion by either phone or by visiting the participants at home. To comply with general data privacy rules, each participant can decide in the consent form whether they want to be contacted by the study nurse.

## Case report form

Physicians together with the participants will complete a Case Report Form (CRF) at each routine visit and participants additionally for each attack. These CRFs differ slightly:

- The CRF for the first visit asks for physical examinations, details of the most recent attack (location of edema, duration), the on-demand and prophylactic treatment, and family history.
- The Follow-Up CRFs ask for details to the most recent attack (location of edema, duration) and the on-demand and prophylactic treatment
- The CRFs filled during the attack sampling ask for the time points when the samples A-F (see Fig. 3) were collected, the symptoms experienced during the attack, and the used on-demand treatment for this attack.

All CRFs will be transferred to a server of CENTOGENE GmbH as an electronic (e)CRF and are stored there with appropriate measures to maintain data privacy regulations (all data are encrypted in transit with TLS1.2). CENTOGENE GmbH operates core services in an external Data Center. Based on multiple certifications, the Data Center provider ensures the compliance to applicable international legislation (e.g. GDPR, HIPPA) and a high level of IT Security and Business Continuity. The Data Center is certified for different quality standards, e.g.:

- ISO 9001—Quality Management System
- ISO 27001—Information Security Management System.

## Genetic + mass spectrometry analysis details

All enrolled participants will be undergoing genetic testing for HAE-1/2 by analyzing the Visit 1 blood samples. For this, a customized amplicon-based next generation sequencing (NGS) approach is used to selectively amplify and enrich all coding regions of the *SERPING1* gene.

Libraries are generated with Illumina compatible adaptors and sequenced on an Illumina platform. An in-house bioinformatics pipeline (CentoMD®) and comprehensive variant filtering are applied. All potential disease-causing variants, including the ones reported in HGMD®, in ClinVar and in CentoMD® are considered. Any relevant variant not meeting the quality control standards will additionally be confirmed by Sanger sequencing.

In cases where no relevant single nucleotide variants are detected by the above described NGS, a Multiplex ligation-dependent probe amplification (MLPA) analysis of the *SERPING1* is conducted for the detection of large deletions/duplications.

All samples (from routine visits and finger pricking) will be analyzed via LC–MS for the concentration of C1-INH, C4 and HKa.

In addition, all samples (from routine visits, finger pricking, healthy and non-HAE disease individuals) will be analyzed using the liquid chromatography technique combined with ion mobility—high resolution mass spectrometry (LC/IM-QToF MS) in an effort to identify novel biomarkers and metabolites. For this, study samples will be prepared and analyzed in an identical manner, obtaining an untargeted mass spectrometric profile containing the following information for all features (peaks or compounds): collision cross section (CCS, Å), accurate molecular weight of the ion (m/z) and retention time (RT). Subsequent exploratory analyses will be focused on identification and characterization of the metabolites of interest.

## Supplementary Information

**Additional file 1.** The file contains a list of all sites that contributed to enrollment of participants.

## Data Availability

To address specific scientific questions anonymized date and/or materials from CENTOGENE repository can be sent on request. The data/materials can be sent to researchers and groups of researchers 12 months after the completion of the HAEKA study. Requests should be sent to Clinical.Studies@centogene.com and are to be decided within four weeks. Positive votes of the Scientific Advisory Board (SAB) as well as the agreement of CENTOGENE which will not refuse these for inequitable reasons, are necessary.
