# Supplementary material for: HAE patient self-sampling for biomarker establishment
Source: Orphanet J Rare Dis. 2021 Sep 28;16:399. doi: 10.1186/s13023-021-02021-x (PMC8478266; doi:10.1186/s13023-021-02021-x)
Supplement: Supplementary file 1 — Additional file 1. The file contains a list of all sites that contributed to enrollment of participants. [file 13023_2021_2021_MOESM1_ESM.pdf]

Initiated and contributing HAEKA centers

Department of Dermatology and Allergy
Charité - Universitätsmedizin Berlin
Berlin
Germany

Medizinische Hochschule Hannover
Klinik für Dermatologie, Allergologie und Venerologie
Hannover
Germany

Klinik für Dermatologie, Venerologie und Allergologie
Universitätsmedizin Leipzig
Leipzig
Germany

HNO-Klinik, Kopf- und Halschirurgie
Universitätsklinikum Ulm
Ulm
Germany

Universitätsklinikum Frankfurt
Klinik für Kinder- und Jugendmedizin
Frankfurt
Germany

HZRM Hämophilie Zentrum Rhein Main GmbH
Mörfelden-Walldorf
Germany

Department of Dermatology and Allergy
Hautklinik und Poliklinik der Universitätsmedizin
Johannes Gutenberg-Universität KöR
Mainz
Germany
